# Estimating technical efficiency of Turkish hospitals: implications for hospital reform initiatives

**DOI:** 10.1186/s12913-018-3239-y

**Published:** 2018-06-04

**Authors:** Mustafa S. Yildiz, Vahé Heboyan, M. Mahmud Khan

**Affiliations:** 1 Ministry of Health, Ankara, Turkey; 2 Department of Clinical and Digital Health Sciences, College of Allied Health Sciences, Augusta University, 987 St. Sebastian Way, EC 4314, Augusta, GA 30912 USA; 3 Department of Health Services Policy & Management, University of South Carolina, Columbia, USA

**Keywords:** Hospital efficiency, Stochastic frontier model, Health transformation program, Public and private hospitals, Turkey

## Abstract

**Background:**

The Government of Turkey has initiated a series of major health reforms in 2003 with an objective of increasing access to health care services and improving efficiency of public and private hospitals. This study attempts to understand the technical efficiency of public and private hospitals in Turkey to better guide hospital reform.

**Methods:**

We use data from 1079 public and private hospitals and translog stochastic production frontier was adopted to estimate technical inefficiency of hospitals.

**Results:**

Results indicate that there is no statistically significant difference in the degree of inefficiency of hospitals by geographic location or its level of economic development. Efficiency scores vary significantly across hospital types with Ministry of Health (MoH) General Hospitals being the most efficient followed by MoH teaching hospitals. Better performance of MoH hospitals may be due to successful implementation of 2003 health reforms in Turkey, which intended to improve resource utilization within and across MoH hospitals. Among MoH hospital types, integrated county hospitals were the least efficient. Since the hospital outcome measure did not include the value of medical training, efficiency scores of university hospitals became relatively low. Wide variability of efficiency scores of private general hospitals implies the existence of both highly efficient and inefficient hospitals in the private sector.

**Conclusions:**

Efficiency differences of various hospital types can be leveraged to guide future reforms by emphasizing the strengths of general hospitals and improving the referral system from county hospitals to general hospitals. Encouraging resource sharing across hospitals, as being done by the 2011 reforms, should further improve hospital efficiency. Promoting private hospitals may not necessarily be efficiency enhancing due to high variability of private hospitals in terms of efficiency scores. Similarly, implementation of common productivity standards and quality control measures are likely to improve hospital technical efficiency scores further.

## Background

Efficiency analysis in health care sector has attracted significant interest in recent decades due to escalating health care costs [1, 2]. Better understanding of health facility efficiency is important for ensuring effective use of health resources, especially in countries where public involvement in health care provision is high. Since public sector health facilities, in many cases, do not compete in the marketplace, alternative strategies must be devised for improving efficiency in resource use [2]. In modern health care system, health sector consists of many different types of facilities and organizations and system-wide efficiency measurement often requires estimation of efficiency for each of the major sectors like insurance providers, hospitals, nursing homes, primary care facilities, etc. [3].

Turkey’s health care system has gone through significant structural changes in the last few decades. In 2015, public expenditure was about 79% of total national health care expenditure of the country [4]. Greater involvement of the government in health sector allowed better coordination of service provision and improved access to services. Turkey also saw very rapid improvements in population health since 1980s. Significant improvements were reported in almost all health outcome measures. Life expectancy at birth has increased from about 65 years in 1990 to 78 years in 2013–15 [5]. However, these accomplishments have not been equally distributed geographically [6] and, despite the rapid improvements, Turkey still lags behind most of the Organization for Economic Cooperation and Development (OECD) countries in terms of health outcomes and health care resource availability (Tables 1 and 2).

**Table 1:** Basic Health Indicators for Turkey and OECD34 averages, 2013 (or nearest year)

Indicator		OECD34 average	Turkey	Rank^a^ (out of 34 OECD countries)
Life expectancy at birth		80.5	76.6	31
Infant mortality (per 1000 live births)		3.8	10.2	34
Total expenditure on health, % GDP		8.9	5.1	34
Total expenditure on health, per capita, US$ PPP		3453	941	34
Physicians, per 1000 population		3.3	1.8	34
Nurses, per 1000 population		9.1	1.8	34
Hospital beds, per 1000 population		4.8	2.7	31
MRI units per 1 million population		14.1	10.5	20^b^
CT scanners per million population		24.4	14.2	25^b^

^a^A lower number indicates higher ranking

^b^Out of 32 OECD countries

Source: OECD [20]

**Table 2:** Geographic variations in health outcomes, availability of health resources and utilization of hospital services in Turkey, 2012

	Centraleast Anatolia	Central Anatolia	Northeast Anatolia	Istanbul	Southeast Anatolia	Aegean	East Marmara	East Black Sea	West Marmara	West Black Sea	West Anatolia	Mediterranean
Perinatal Mortality per 1000 live birth	10.9	7.1	10.9	6.7	11.3	7.5	7.5	7.0	7.8	8.4	6.7	7.6
Neonatal Mortality per 1000 live birth	6.5	3.9	6.3	3.2	6.2	3.5	3.7	3.8	3.9	3.9	3.0	3.7
Post-neonatal Mortality per 1000 live birth	4.6	3.3	4.1	2.2	4.4	2.3	2.2	3.0	2.5	3.0	2.5	3.3
Mortality under 5 years per 1000 live birth	16.7	10.8	13.9	8.2	16.1	8.7	9.0	9.9	9.1	9.9	8.3	10.8
Maternal Mortality per 1000 live birth	25.5	25.9	32.2	15.1	14.7	13.6	7.7	21.5	7.8	17.2	12.7	10.7
Poverty rate (%), 50% poverty risk threshold	13.4	12.4	13.2	9.6	12.8	11.6	10.8	11.1	13.0	12.0	12.9	13.7
Number of hospital beds per 10,000	26.7	28.4	28.8	23.3	19.7	27.2	26.1	33.1	27.4	30.3	35.8	23.8
Number of ICU beds per 10,000	2.9	2.9	2.0	3.3	3.1	3.1	3.0	2.9	2.4	2.8	3.8	3.3
Per Capita visits to hospitals	4.5	4.9	4.7	4.2	4.1	4.9	4.8	5.8	5.0	5.4	5.0	4.6
Physicians per 100,000 population	144	159	142	192	121	183	156	159	150	152	266	155
Nurses per 100,000 population	257	277	253	191	184	279	254	339	278	296	302	242
Surgical operations per 1000 population	49.5	63.4	57.2	56.3	49.6	58.0	59.1	59.8	44.4	53.6	73.8	64.7
Surgical operations per 1000 population (Group A)^a^	3.1	5.0	3.5	6.8	2.8	6.4	6.8	6.0	3.5	5.4	8.9	5.5
Number of MRI exams per 1000 population in hospitals	31.8	19.9	23.4	28.0	24.5	22.5	22.9	25.8	18.7	25.8	24.8	23.0
Bed Occupancy Ratio	65.4	60.8	65.0	70.1	67.9	67.5	67.8	62.2	67.8	62.2	68.5	68.6

Source: General Directorate of Health Research [22]

^*^Surgical operations are classified into groups A to E based on the severity of the operations

In 2003, the Government of Turkey initiated a set of major health reforms, the Health Transformation Program (HTP), with an objective of increasing access to health care services and improving efficiency of hospitals through (i) implementation of General Health Insurance (GHI), (ii) establishing autonomous hospital structures, (iii) improving qualifications of health professionals and their work motivation, and (iv) deployment of an effective health information system [7]. The reforms integrated social security schemes under the Social Insurance Institution (SII), transferred public hospitals from the insurance agency to the Ministry of Health (MoH), instituted a performance-based supplementary payment system (P4P), and implemented family medicine model of health care delivery [8].

These reforms gave the Ministry and newly established Public Hospital Administration of Turkey (PHAT) the authority to align hospital mission, goals, and objectives with national priorities in health. Since the government became the major source of funding, the MoH could influence and manage use of resources (in both public and private hospitals) and implement more effectively health care service standards.

This research is an attempt to understand efficiency of hospital sector in the provision of services in Turkey. The analysis assumes that efficiency of any production unit is affected by its specific goal and objectives and therefore, factors affecting efficiency will be different for different hospital types. For empirical analysis, hospitals in Turkey were grouped into categories based on ownership (MoH, university, private) and teaching status/type (teaching, general, integrated). No single health policy can be equally effective in improving resource allocation in all these different hospital types. Therefore, it is important to understand the relative efficiency in healthcare resource utilization for each of the hospital types so that the MoH can develop targeted policy options.

The primary objective of this study is to estimate technical inefficiency of Turkish hospitals and to analyze the role of various hospital-specific and region-specific factors affecting the efficiency scores. The adoption of health care service standards and alignment of goals and objectives of all hospitals with national priorities should reduce variability of efficiency levels. For estimating the efficiency scores, this study uses hospital data of MoH Health Services General Directorate. Stochastic Frontier Analysis (SFA) approach of Aigner, Lovell, and Schmidt [9] and Meeusen and van der Broeck [10] were used for estimating the efficiency scores and single-step estimation approach suggested by Battese and Coelli [11, 12] (discussed in detail in section “Methods”) was applied to identify the factors affecting efficiency.

A number of studies have attempted to estimate efficiency of Turkish hospitals but most focused on either a single hospital category [13–15] and/or a small subset of hospitals [16, 17], primarily utilizing Data Envelopment Analysis (DEA). For example, Sahin et al. [15] analyzed the operational performance of the MoH general public hospitals in the aftermath of HTP. Authors indicate that the HTP reforms improved hospital productivity during 2005–08. Narci et al. [18] examined the competition and technical efficiency among public and private general hospitals in Turkey. Results showed that only 17% of these hospitals were technically efficient, but they did not observe any statistically significant relationship between market competition and efficiency. None of the reviewed studies examined relative efficiency of hospitals by considering *all* the public and private facilities taken together. Moreover, recent health sector reform initiatives are supposed to improve hospital efficiency and the analysis with recent data should be able to indicate how the hospital efficiency has changed over the years.

This study is the first in Turkey that analyzes efficiency of the hospital sector by using information on *all* general hospitals, both public and private. In addition, this study has made an attempt to link health sector reform policies and hospital efficiency. In terms of estimation technique, this paper adopts an empirical approach to account for the ‘zero-value’ problem in production function analyses (modified production function) and uses the simultaneous estimation of efficiency scores and determinants of efficiency to obtain unbiased estimates.

The paper is structured as follows. Section “Health system in Turkey” provides a brief overview of the health system in Turkey. Section “Methods” describes the methodology, and model specification. Sources of data are presented in section “Results”. The results are presented in section “Discussion” and concluding remarks and policy recommendations are provided in Section “Conclusions”.

## Health system in Turkey

Turkish health system has gone through rapid changes since the adoption of Health Transformation Program (HTP) in 2003 which was designed to change delivery of services, financing of the system, organizational set-up, level of health expenditure, health infrastructure, and mechanism of resource allocation. The improvements in health outcomes and health facility performance in recent years are often attributed to the strategies and policies implemented under the HTP [19].

One principal objective of the HTP was to address the issues related to fragmentation of health care provision and financing. Two governmental agencies became responsible for provision and financing of health care. At the national level, General Health Insurance Scheme (GHIS) was introduced in 2008 which now covers 99.5% of population. Turkey had the second lowest private health insurance coverage (5.6% in 2013) among all the OECD countries [20]. Provision of healthcare services is primarily controlled by the MoH including the Ministry of Defense (MoD) health facilities that were recently transferred to the MoH management. Private providers are integrated into the system through contractual agreements with the social health insurers [6].

For empirical analysis, we have categorized hospitals in Turkey based on ownership, teaching status, size and scope of services rendered. If ownership is used for categorization, for 2012, hospitals in Turkey (1483 total) can be grouped into MoH hospitals (832 hospitals), university hospitals (65 hospitals), private hospitals (541 hospitals), MoD hospitals (42 hospitals), and local administration hospitals (3 hospitals). The scale and scope of services rendered are also different among the hospital types with significant geographic variability (Table 2) ([21], p., 143).

The MoH hospital category can further be subdivided into: MoH teaching hospitals, responsible for residency training and tertiary level care, the MoH general hospitals, providing secondary level care with intensive care units and emergency services and integrated hospitals which provide limited essential patient care services in low population-density rural areas in partnership with local general hospitals.

Private university hospitals primarily provide medical education and training for residents, while the private hospitals serve the secondary and tertiary level needs of population in their catchment areas. Public university hospitals also serve tertiary needs of the population in addition to medical training and teaching responsibilities. Specialty hospitals, both public and private, have specialized focus such as emergency and traumatology, physical therapy and rehabilitation, chest and cardiovascular diseases, ophthalmology, obstetric and child health, cardiology, etc.

A number of reform initiatives were adopted in Turkey since 2003 within the HTP framework. Since the beginning of HTP, the MoH has been successful in expanding health service delivery and quality [19] with significant investments in (i) new infrastructures for providing better quality health services (e.g. new hospitals), (ii) medical technologies (e.g. total number of computerized tomography and magnetic resonance devices), (iii) increasing number of beds and intensive care unit beds, (e.g. intensive care unit beds in MoH hospitals increased from 869 in 2002 to 10,321 in 2012), and (iv) increasing availability of medical personnel (e.g. number of Specialist Physicians increased from 45,457 to 70,103 and nurses increased from 72,393 to 134,906 over 2002 to 2012) [22].

As part of the wider health system reform, hospital service coordination was decentralized to give local authorities financial and administrative autonomy [23]. The reform has reorganized the MoH and rural hospital structure by uniting 843 MoH hospitals into 87 Public Hospital Unions (PHUs) and devolved important tasks to these PHUs in 2012. The PHAT was delegated the authority of establishing financial and administrative regulations for public hospitals and carrying out annual monitoring and assessment of public hospital and PHUs for improving effectiveness, quality, and efficiency [23].

As a result of this reorganization, the MoH assumed the responsibility of preparing and implementing hospital service delivery standards (public, university, and private hospitals) and human resource planning for the entire health system. In addition, MoH approves private hospital start-ups and determines quotas of doctors and their specialties for the private sector [23]. To better understand the effect of these policy changes on efficient use of resources, it is important to estimate relative efficiency of hospitals by ownership, size and geographic location.

Consistent with health sector reform policies, public health expenditure as share of national health expenditure increased from 68.1% in 2001 to 74.9% in 2011. Although the public funding for health care has not reached the OECD average yet, such a rapid increase in public funding reflects significant injection of new resources in the health sector. General government expenditure on health as a percentage of total government expenditure has increased from 9.5% in 2001 to 12.8% in 2011. Total expenditure on health as a percentage of Gross Domestic Product (GDP) has also increased from 5.2 to 6.7% over 2001 to 2011 (Table 3).

**Table 3:** Changes in health care financing in Turkey, 1995–2011

№	Indicator	1995–2000	2001	2002	2003	2004	2005	2006	2007	2008	2009	2010	2011
1	Total expenditure on health as a percentage of gross domestic product	4.13	5.16	5.36	5.34	5.37	5.45	5.81	6.04	6.07	6.75	6.67	6.66
2	General government expenditure on health as a percentage of total government expenditure	10.52	9.54	9.07	9.73	10.75	11.28	11.95	12.13	12.79	12.79	12.79	12.79
3	General government expenditure on health as a percentage of total expenditure on health	67.85	68.07	70.68	71.92	71.25	67.84	68.34	67.83	73.02	75.14	74.79	74.94
4	Private expenditure on health as a percentage of total expenditure on health	32.15	31.93	29.32	28.08	28.75	32.16	31.66	32.17	26.98	24.86	25.21	25.06
5	External resources for health as a percentage of total expenditure on health	0.80	0.27	0.01	0.12	0.01	0.01	0.03	0.09	0.07	0.01	0.00	..
6	Out-of-pocket expenditure as a percentage of private expenditure on health	91.42	71.55	67.68	65.75	66.91	70.78	69.4	67.82	64.41	64.41	64.41	64.41
7	Private prepaid plans as a percentage of private expenditure on health	..	..	..	..	5.63	5.91	5.99	6.11	7.31	7.31	7.31	7.31
8	Social security expenditure on health as a percentage of general government expenditure on health	43.55	54.46	57.51	59.91	60.85	56.08	56.63	54.44	57.04	57.04	57.04	57.04

Source: WHO [4]

“..”: data not available

## Methods

### Estimation methodology

In the economics literature, there are two broad categories of analytic approaches to estimate the cost or production frontiers and associated efficiencies: parametric and non-parametric methods. The former uses econometric approaches to estimate the functional forms and the latter uses observed data to estimate the frontier without placing conditions on the functional form [2]. The Stochastic Frontier Analysis (SFA) and DEA are the most prominent forms of the parametric and non-parametric approaches, respectively. Both of these approaches have their strengths and weaknesses and the empirical literature has used both approaches without a clear argument for either approach. Jacobs et al. [2] provide a detailed individual and comparative examination of these approaches. In this study, we are adopting the SFA approach to address the research objectives and also provide comparative basis for studies utilizing alternative approaches.

Production function analysis implicitly assumes that all firms, on the average, are technically efficient and the average production function reflects the underlying technical efficiency. However, Kumbhakar and Lovell [24] suggested that “*not all producers are technically efficient*” and, therefore, it becomes desirable to move away from traditional average production functions to frontiers (p. 3). The *production frontier* defines the maximum output that can be produced with available inputs at a given technology (or the minimum inputs required to produce the outputs with a given technology). Technically efficient producers operate on their production frontier, whereas those who operate below the frontier are labeled as inefficient. The econometric implication of such reformulation is the decomposition of the error term into a traditional symmetric random noise and a new inefficiency component ([24], p. 42).

There are two classes of econometric techniques used for efficiency analysis: corrected ordinary least squares (COLS)^1^ and stochastic frontier analysis (SFA). The latter is based on the specification of a stochastic production frontier proposed by Aigner et al. [9] and Meeusen and van der Broeck [10]. It allows the firms to be technically inefficient relative to their own frontier rather than to some norm. This alleviates the concerns associated with the estimation of deterministic production frontiers where the parameters are computed rather than estimated, making hypothesis testing impossible [25]. Both COLS and SFA are specified by the general production frontier of the form [26]:

1 $$ \ln {y}_i={\mathrm{x}}_i^{\hbox{'}}\beta -{u}_i $$

where, *y*_*i*_ is the output of the *i*^th^ firm; the x_*i*_ is a *Kx1* vector containing the logarithms of inputs; *β* is a vector of unknown parameters; and *u*_*i*_ is a non-negative random variable representing technical inefficiency.

The difference between COLS (and its variants) and SFA is in their interpretation of the error term; COLS assumes that the entire error term is the inefficiency and SFA assumes that the error term is a combination of a random error term and an inefficiency term [2]. Therefore, in the presence of inefficiency and random shocks (*υ*_*i*_), empirically estimable frontier production function can be written as:

2 $$ \ln {y}_i={\mathrm{x}}_i^{\hbox{'}}\beta +{v}_i-{u}_i $$

or

3 $$ {\displaystyle \begin{array}{l}\ln {y}_i={\beta}_0+\sum \limits_{j=1}^k{\beta}_j\ln {x}_{ji}+{v}_i-{u}_i\kern2.319995em \left(\mathrm{Cobb}\hbox{-} \mathrm{Douglas}\right)\\ {}\ln {y}_i={\beta}_0+\sum {\beta}_j\ln {x}_{ji}+\frac{1}{2}\sum \limits_{j=1}^k\sum \limits_{h=1}^k{\beta}_{jh}\ln {x}_{ji}\ln {x}_{hi}+{v}_i-{u}_i\kern1.44em \left(\mathrm{Translog}\right)\end{array}} $$

where, (*v*_*i*_ - *u*_*i*_) is the decomposed error term in which *v*_*i*_ allows for randomness across firms and captures the effect of measurement error, other statistical noise, and random shocks outside the firm’s control and *u*_*i*_ captures the effect of inefficiency [27].

This study adopts the stochastic frontier approach with one-stage simultaneous estimation strategy suggested by Battese and Coelli [11, 12] to estimate the technical efficiency scores for Turkish public and private hospitals. An extensive review of SFA applications to hospitals (in US) can be found in Rosko and Mutter [28].

Battese and Coelli [12] note that most theoretical stochastic frontier production functions do not explicitly model the technical inefficiency effects using appropriate explanatory variables, which usually are neither output nor input variables. To address this concern, we specify a model with inefficiency term (mean μ_i_ and variance *σ*^2^) as the dependent variable and **z**_m_, a set of variables affecting technical inefficiency. The last term, *ω*_i_ represents the stochastic error term (Eq. 3).

4 $$ {\mu}_i={\gamma}_0+\sum {\gamma}_{\mathrm{m}}{\mathrm{z}}_{\mathrm{m}}+{\omega}_{\mathrm{i}} $$

Estimation of stochastic frontier requires specifying the distributional characteristics of both components of the residual. It is commonly assumed that *v*_*i*_ is normally distributed with zero mean and constant variance. Jacobs et al. ([2], p. 54–56) suggests that inefficiency estimates are sensitive to the choice of distribution for *u*_*i*_ and no economic criteria are available to guide this choice. A review of recent literature on SFA by Rosko and Mutter [28] has showed that, in both general and health care literature, SFA results have been found to be robust across distributional assumptions on inefficiency term. Furthermore, considering the critique of Newhouse [29] and remedy proposed by Stevenson [30], Rosko [31] concluded that the assumption of truncated normal distribution appear appropriate for the inefficiency term. Following Battese et al. [32] and Coelli and Battese [33], we assume that the inefficiency term *u*_*i*_ follows truncated (at zero) normal distribution with mean *μ*_*i*_ and variance *σ*^2^.

In the empirical work, most researchers have opted for estimation of production frontier (Eq. 3) and inefficiency effects (Eq. 4) in a two-stage approach, where the first stage involves estimation of the stochastic frontier and the second stage estimates factors affecting technical inefficiency. This approach, Battese and Coelli [12] argued, violates the identically distributed assumption of inefficiency effects in the stochastic frontier model. Kumbhakar et al. [34], Reifschneider and Stevenson [35], Huang and Lui [36], and Battese and Coelli [12] proposed single-stage, simultaneous estimation of the parameters. “*This one-stage approach is less objectionable from a statistical point of view and is expected to lead to more efficient inference with respect to the parameters involved*” ([33], p., 105). For empirical modeling, this paper has used single-stage simultaneous estimation approach.

### Zero-value problem

Often production functions involve explanatory variables that have zero values making logarithmic transformations of production functions impossible. For example, in a health center, nurses and other paramedics may provide health care services without the presence of any physician or a hospital may not have some particular equipment (e.g. an x-ray machine or CT scan). Battese [37] and Battese et al. [32] argue that confining the analysis to those who utilize positive amounts of inputs may not be the most appropriate method of estimation as it implies excluding producers from the analysis with at least one zero input value.

The so-called ‘zero-value’ problem in estimation of production functions has been addressed in various ways. Some have suggested assigning an arbitrary small value to the zero-value, while others have tried to fit other functional forms that did not violate the zero input levels such as quadratic equation model. Moss [38] argues that the former is conditioned by the choice of the small number, while the latter is unacceptable from a purely theoretical perspective and has implications for global concavity of the production function. If the cases with zero-values are substantial in the sample (Battese [37]), substituting with an arbitrary small value may result in biased estimates. The recommendations are either to use bootstrapping to construct an alternative sample or to use dummy variable associated with zero-value observations to generate unbiased estimates for the production functions. This study adopts the approach by Battese [37], where the input variable that contains zero values is modified as:

5 $$ {x}_i^{\ast }=\ln \left(\max \left[{x}_i,{D}_i\right]\right) $$

where, *x*_*i*_ is the *i*^th^ explanatory variable that contains zero-value observations and *D*_*i*_ is defined as:

6 $$ {D}_i=\left\{\begin{array}{l}1\; if\;{x}_i=0\\ {}0\; if\;{x}_i>0\;\end{array}\right. $$

### Data source and empirical model specification

#### Data

This study has used cross-sectional data obtained from the MoH Health Services General Directorate of Turkey for the year 2012. The data set consists of a comprehensive sample of 1394 hospitals (843 MoH hospitals, 62 University hospitals and 489 private hospitals). Since the purpose of the analysis is to estimate efficiency scores for acute care general hospitals, 93 specialty hospitals were dropped from the data set. Some of the hospitals in the data set had no beds at all and these hospitals were also dropped (134 hospitals) and finally 88 hospitals were dropped for substantial missing data. Therefore, the final dataset had 1079 hospitals with different ownerships (398 private, 56 university, and 625 MoH hospitals) and types (98 teaching and 981 general).

#### Functional form

The literature identifies the Cobb-Douglas and translog as the two leading functional forms employed in the literature to specify and estimate production functions in hospital inefficiency studies [39–41]. They both have their own merits and drawbacks. Some researchers are in favor of using the translog functional form, especially for larger samples, while others support the use of Cobb-Douglas functional form [39, 40, 42, 43]. We have performed the generalized likelihood ratio test^2^ to identify the proper functional form to use for our data. The test results^3^ reject the null hypothesis that Cobb-Douglas is the appropriate model to use at 0.05 level of significance, implying that the translog functional form is more suitable for our analysis. Therefore, the production frontier is empirically modeled as a modified translog function that accounts for the zero-value problem. The function is presented below:

7 $$ {\displaystyle \begin{array}{c}\ln \left({output}_i\right)={\beta}_0+{\beta}_1\ln bed+{\beta}_2\ln clinicians+{\beta}_3\ln doctors+{\beta}_4\ln {devices}^{\ast }+\\ {}+{\beta}_5\ln {icubeds}^{\ast }+{\beta}_6\ln admin+{\beta}_7 devices\_d+{\beta}_8 icubeds\_d+\\ {}+{\beta}_{11}0.5{\left(\ln bed\right)}^2+{\beta}_{22}0.5{\left(\ln clinicians\right)}^2+{\beta}_{33}0.5{\left(\ln doctors\right)}^2+\\ {}+{\beta}_{44}0.5{\left(\ln devices\right)}^2+{\beta}_{55}0.5{\left(\ln icubeds\right)}^2+{\beta}_{66}0.5{\left(\ln admin\right)}^2+\\ {}+{\beta}_{12}\left(\ln bed\times \ln clinicians\right)+{\beta}_{13}\left(\ln bed\times \ln doctors\right)+{\beta}_{14}\left(\ln bed\times \ln devices\right)+\\ {}+{\beta}_{15}\left(\ln bed\times \ln icubeds\right)+{\beta}_{16}\left(\ln bed\times \ln admin\right)+{\beta}_{23}\left(\ln clinicians\times \ln doctors\right)+\\ {}+{\beta}_{24}\left(\ln clinicians\times \ln devices\right)+{\beta}_{25}\left(\ln clinicians\times \ln icubeds\right)+\\ {}+{\beta}_{26}\left(\ln clinicians\times \ln admin\right)+{\beta}_{34}\left(\ln doctors\times \ln devices\right)+{\beta}_{35}\left(\ln doctors\times \ln icubeds\right)+\\ {}+{\beta}_{36}\left(\ln doctors\times \ln admin\right)+{\beta}_{45}\left(\ln devices\times \ln icubeds\right)+{\beta}_{46}\left(\ln devices\times \ln admin\right)+\\ {}+{\beta}_{56}\left(\ln icubeds\times \ln admin\right)+ error\end{array}} $$

where β_0_ is the intercept; β_1_, β_2_, β_3_, β_4_, β_5_, β_6_, β_7_, and β_8_ are the first order derivatives; β_11_, β_22_, β_33_, β_44_, β_55_, and β_66_ are the second order derivatives; and β_12_, β_13_, β_14_, β_15_, β_16_, β_23_, β_24_, β_25_, β_26_, β_34_, β_35_, β_36_, β_45_, β_46_, and β_56_are cross second order derivatives. Since Eq. 7 is in double log form, the estimated coefficients are the elasticities between dependent and independent variables.

When using the translog functional form, to truly assess the effect of each input, the marginal effects of inputs are of interest rather than the values of the input coefficients. We calculate the marginal effects for each input using the following equation:

8 $$ {e}_j=\frac{\partial \ln (y)}{\partial \ln \left({x}_j\right)}={\beta}_j+\sum \limits_{j=1}^k\sum \limits_{h=1}^k{\beta}_{jh}\ln {x}_h $$

The technical inefficiency term for this model is estimated as (using Eq. 3):

9 $$ {\mu}_i={\gamma}_0+{\gamma}_1(type)+{\gamma}_2(income)+{\gamma}_3(region)+\omega $$

Apriori expectations are that general hospitals (*type*) will have higher efficiency than the teaching hospitals and the MoH hospitals will have higher efficiency scores than non-MoH hospitals. The level of the economic development (*income*) in the province where the hospital is located is hypothesized to affect the inefficiency because socio-economic and cultural characteristics affect access and utilization of healthcare services. Finally, regional differences (*region*) are likely to affect hospital efficiency due to specific spatial factors. Efficiency scores among MoH hospital types or between MoH and private hospitals will help policy makers to identify possible interventions in order to improve resource use of hospitals. The analysis will also be able indicate whether increased market competition by encouraging establishment of private hospitals will help improve efficiency of hospital sector in general.

For comparative analysis of efficiency scores, hospitals should be compared with the most efficient hospitals within the sample. Although the size of the hospitals may be associated with different scale and scope of health services, comparing relative efficiency of hospitals with the corresponding efficiency frontier should not bias the results. Small size hospitals are compared with the efficient units within the same size groups as the production function identifies the most efficient outcomes for all hospital sizes in the sample. Therefore, estimating one production function should not necessarily be a problem unless significant part of outputs were not measured in the data set.

In Eq. 7, the **dependent variable** (*output*) is a measure of aggregate hospital output which was derived by using Eq. 10. Eq. 10 aggregates multiple outputs of hospitals using output-specific weights, the average market prices, *p*, of hospital services. Since public funding is such a big component of hospital expenditure, the prices of hospital services set by the SSI [44] are considered the relevant prices to use to derive the measure of aggregate output.

10 $$ {\displaystyle \begin{array}{l}{output}_i=\sum \limits_{j=1}^5\left({surgeries}_{ij}\times {p}_j\right)+\sum \limits_{q=1}^7\left({beds}_{iq}\times {rate}_{iq}\times 365\times {p}_q\right)+{inpatient}_i\times {p}_{inpatient}\\ {}+\sum \limits_{m=1}^3\left({delivery}_{im}\times {p}_m\right)+\sum \limits_{l=1}^5\left({tech}_{il}\times {p}_l\right)+\sum \limits_{h=1}^2\left({visits}_{ih}\times {p}_h\right)\end{array}} $$

Price index for each output type is generated by assigning a base value of 1.00 to the least expensive transaction/output – doctor, ER, and inpatient bed prices. This aggregation approach allows estimation of a one output frontier production function for multi-output production units.

**Independent variables** in Eq. 7 are related to hospital infrastructure, technology, and human resources. Tables 4 and 5 list the variables used in empirical modelling with associated summary statistics. In our sample, variables *icubeds* and *devices* have 316 (29.3%) and 4 (0.4%) observations with zero values, respectively. Even though variable *devices* does not have ‘substantial’ number of observations with zero value, to ensure consistency in our modeling approach, we modified both variables using the approach indicated in Eq. 5 to address the ‘zero-value’ problem.

**Table 4:** Description of variables used in empirical models and summary statistics

Variable	Description	Mean	SD	Min	Max
Variables in Frontier Model					
*output*	Hospital composite output (see Eq. 9)	14.3 M	29.0 M	2885	287 M
*bed*	Number of inpatient hospital beds	144	215	2	1816
*doctors*	Number of doctors except residents	55	83	1	1030
*clinicians*	Number of non-doctor health professionals (nurses, midwives, technicians, etc.)	174	231	7	2006
*devices*	Number of X-Ray, MR, CT, ECG, Doppler	12	17	0	458
*devices_d*	Dummy to control for *zero-value* problem				
*admin*	Number of administrative staff	39	53	1	489
*icubeds*	Intensive Care Unit (ICU) Beds	17	25	0	230
*icubeds_d*	Dummy to control for *zero-value* problem				
Variables in Inefficiency term model					
*type*	Hospital ownership type (%)				
	MoH General	42.02			
	MoH Integrated County Hospitals	11.97			
	MoH Teaching	3.99			
	Private General	36.83			
	Private University	1.21			
	Public University	3.99			
*income*	Per capita income proxied by Regional per capita Gross Domestic Product				
	Less than $5000	4.36			
	$ 5001–10,000	12.64			
	$ 10,001–15,000	28.94			
	$ 15,001–20,000	39.33			
	$ 20,001 and above	13.73			
*region*	Statistical regions and percent of hospitals in each region				
	Istanbul	15.31			
	West Marmara	5.94			
	Aegean	12.8			
	East Marmara	8.91			
	West Anatolia	8.91			
	Mediterranean	11.41			
	Central Anatolia	5.94			
	West Black Sea	7.88			
	East Black Sea	4.92			
	North East Anatolia	3.53			
	Central East Anatolia	6.22			
	South East Anatolia	8.26

**Table 5:** Summary statistics for output elements and corresponding price indices

Variable	Description	Mean	SD	Min	Max	Price index
*operations*	Number of annual surgical operations^a^					
	Type A	332	667	0	6485	266.53
	Type B	1190	1887	0	14,757	75.73
	Type C	1797	2490	0	20,611	37.60
	Type D	1639	2776	0	25,107	23.13
	Type E	2921	7284	0	109,748	11.07
*icubeds*	Number of beds in the ICU units					
	Adult level 1	70,254	163,681	0	1,617,388	16.67
	Adult level 2	75,591	175,739	0	2,385,640	30.00
	Adult level 3	117,145	316,273	0	3,403,698	50.00
	Neonatal level 1	16,690	64,281	0	903,375	16.67
	Neonatal level 2	28,028	95,935	0	1,151,502	30.00
	Neonatal level 3	52,077	160,709	0	1,292,319	50.00
	Pediatric	11,885	66,458	0	949,000	30.00
*inpatient*	Number of inpatient-day (inpatient bed) utilization at each hospital.					
		33,072	58,193	0	470,287	1.00
*Rate*	Average percentage of daily occupancy rates of the beds at the ICU units.					
	Adult	32.18	34.07	0	100	na
	Neonatal	19.22	30.95	0	100	na
	Pediatric	3.39	15.79	0	100	na
*delivery*	Number of annual child deliveries					
	Normal	328	589	0	7381	6.67
	Operation	22	107	0	1595	12.00
	C-section	396	527	0	4692	12.00
*Tech*	Number of annual utilization of the diagnostics equipment					
	ECG	365	601	0	5352	4.80
	MR	653	1047	0	7371	4.80
	BT	701	1244	0	10,220	4.00
	Doppler	2120	6172	0	180,153	2.00
	X-ray	4044	6324	0	54,205	1.00
*Visits*	Number of annual visits to					
	ER	75,351	90,152	0	736,292	1.00
	Doctors	267,580	320,679	1685	2,405,443	1.00

“na” - not applicable

^a^Surgical operations are classified into groups A to E based on the severity of the operations

## Results

The maximum likelihood estimates (MLE) of the logarithmic modified translog stochastic production frontier and inefficiency effects are presented in Tables 6 and 8, respectively. Table 6 reports the set of parameters that explain the impact of production factors on healthcare output. Results show that the number of non-doctor health professionals (ln*clinicians), ICU beds (*ln*icubeds)*, and administrative staff (ln*admin*) are statistically significant at 5% level and have the expected positive sign. ICU beds had the largest impact on the output followed by clinicians and administrative staff. The dummy variable controlling for the ‘zero value’ problem in *devices* is statistically significant and negative implying that hospitals without “devices” exhibit lower outputs.

**Table 6:** Frontier estimation results – translog production function

Variable	Coef.	Std. Error	Z	P > z	95% Conf. Interval	
Frontier Model: dependent variable = log(*output*)						
Inpatient hospital beds (*log*)	−0.3904	0.2548	−1.5300	0.1250	−0.8899	0.1090
Non-doctor clinicians (*log*)	1.1531	0.3090	3.7300	0.0000	0.5474	1.7587
Doctors (*log*)	− 0.4522	0.2831	−1.6000	0.1100	−1.0071	0.1027
Devices (*log*)	0.2977	0.2337	1.2700	0.2030	−0.1602	0.7557
Devices dummy (*=1 if no device*)	−0.6614	0.2165	−3.0600	0.0020	−1.0857	− 0.2372
ICU beds (*log*)	2.5584	0.1710	14.9600	0.0000	2.2232	2.8937
ICU beds dummy (*=1 if no beds*)	−0.0167	0.1205	−0.1400	0.8900	−0.2528	0.2194
Administrative staff (*log*)	0.3217	0.1278	2.5200	0.0120	0.0713	0.5722
ln*bed*^2^	0.0328	0.0436	0.7500	0.4510	−0.0526	0.1183
ln*clinicians*^2^	−0.1171	0.0752	−1.5600	0.1190	−0.2645	0.0302
ln*doctors*^2^	0.0903	0.0726	1.2400	0.2130	−0.0519	0.2325
ln*devices*^2^	0.0221	0.0270	0.8200	0.4130	−0.0308	0.0750
ln*icubeds*^2^	0.1862	0.0259	7.1800	0.0000	0.1354	0.2371
ln*admin*^2^	0.0194	0.0196	0.9900	0.3210	−0.0189	0.0578
ln*bed* × ln*clinicians*	0.1086	0.1035	1.0500	0.2940	−0.0942	0.3113
ln*bed* × ln*doctors*	−0.1246	0.1150	−1.0800	0.2790	−0.3500	0.1009
ln*bed* × ln*devices*	−0.0157	0.0941	−0.1700	0.8670	−0.2002	0.1687
ln*bed* × ln*icubeds*	−0.0876	0.0578	−1.5200	0.1290	−0.2008	0.0256
ln*bed* × ln*admin*	0.0598	0.0542	1.1000	0.2700	−0.0464	0.1660
ln*clinicians* × ln*doctors*	0.2428	0.1102	2.2000	0.0280	0.0269	0.4587
ln*clinicians* × ln*devices*	−0.0267	0.1076	−0.2500	0.8040	−0.2377	0.1842
ln*clinicians* × ln*icubeds*	−0.2386	0.0606	−3.9400	0.0000	−0.3574	−0.1198
ln*clinicians* × ln*admin*	−0.2112	0.0566	−3.7300	0.0000	−0.3221	−0.1002
ln*doctors* × ln*devices*	−0.1513	0.1005	−1.5100	0.1320	−0.3483	0.0457
ln*doctors* × ln*icubeds*	−0.1510	0.0571	−2.6400	0.0080	−0.2629	−0.0391
ln*doctors* × ln*admin*	0.0583	0.0691	0.8400	0.3990	−0.0771	0.1938
ln*devices* × ln*icubeds*	0.0362	0.0457	0.7900	0.4280	−0.0533	0.1257
ln*devices* × ln*admin*	0.1196	0.0521	2.3000	0.0220	0.0176	0.2216
ln*icubeds* × ln*admin*	−0.0522	0.0327	−1.6000	0.1100	−0.1162	0.0118
*Constant*	8.5814	0.5541	15.4900	0.0000	7.4953	9.6675

Only the second order coefficient for ICU beds was statistically significant and combined with statistically significant and positive first order ICU beds coefficient, implies that hospitals investing in additional ICU beds will be able to generate output at an increasing rate. Several interaction terms were statistically significant indicating that the usage levels of the inputs are inter-dependent on each other.

*Output elasticities* of each of the input variables at their mean values were calculated using Eq. 8 and reported in Table 7. The estimates were − 0.04, 0.20, 0.33, 0.12, 1.18, and 0.06 for beds, clinicians, doctors, devices, ICU beds, and staff, respectively. All, except beds were statistically significant at 1% level. ICU beds appear to be the most important factor in the hospital production and exhibits increasing returns to scale (RTS), while all other statistically significant inputs exhibit decreasing returns to scale. Next, in order of importance, are the doctors and clinicians (e.g. nurses). Overall, hospitals in the sample exhibit increasing RTS; a 1% increase in all inputs would increase hospital production by 1.9%.

**Table 7:** Output elasticities of input variables

Inputs	Coef.
Inpatient hospital beds	−0.0399
Non-doctor clinicians	0.2036^a^
Doctors	0.3308^a^
Devices	0.1212^a^
ICU beds	1.1816^a^
Administrative staff	0.0646^a^
Total	1.8618

^a^individual input coefficients significant at 1%

Parameter estimates for the inefficiency term are presented in Table 8. Results indicate no statistically significant difference in the inefficiency of the hospitals by level of economic development (*income*) of the locality. Geographically (*region*), only two regions, West Anatolia and Central East Anatolia, show statistically different (lower) efficiencies compared to the reference group of the Istanbul region. Meanwhile, significant differences, as expected, are present for various hospital types. For example, the MoH General Hospitals are found to be the most efficient hospital type.

**Table 8:** Frontier estimation results – inefficiency equation

Inefficiency term model	Coef.	Std. Error	Z	*P* > z	95% Conf. Interval	
Hospital type (*reference = MoH General Hospitals*)						
MoH Integrated County	−0.3087	1.0999	−0.2800	0.7790	−2.4644	1.8471
MoH Teaching	−10.2968	6.2776	−1.6400	0.1010	−22.6007	2.0070
Private General	5.7161	1.5593	3.6700	0.0000	2.6598	8.7723
Private University	5.3802	1.7796	3.0200	0.0030	1.8923	8.8682
Public University	1.9877	1.3731	1.4500	0.1480	−0.7036	4.6790
Provincial per capita income (*reference = less than $5000*)						
$ 5001–10,000	1.1581	1.4513	0.8000	0.4250	−1.6864	4.0026
$ 10,001–15,000	0.1728	1.0065	0.1700	0.8640	−1.7999	2.1455
$ 15,001–20,000	0.2287	1.1706	0.2000	0.8450	−2.0657	2.5231
Over $20,000	0.7793	1.2776	0.6100	0.5420	−1.7247	3.2834
Regions (*reference = Istanbul*)						
West Marmara	0.0169	0.8593	0.0200	0.9840	−1.6673	1.7010
Aegean	0.3144	0.7433	0.4200	0.6720	−1.1424	1.7712
East Marmara	0.9828	0.7174	1.3700	0.1710	−0.4232	2.3888
West Anatolia	1.3406	0.6433	2.0800	0.0370	0.0798	2.6014
Mediterranean	−0.5174	0.6444	−0.8000	0.4220	−1.7805	0.7456
Central Anatolia	1.5293	0.9611	1.5900	0.1120	−0.3543	3.4130
West Black Sea	−0.0208	1.0204	−0.0200	0.9840	−2.0209	1.9792
East Black Sea	−2.3229	1.7857	−1.3000	0.1930	−5.8228	1.1770
North East Anatolia	−0.2497	1.7456	−0.1400	0.8860	−3.6711	3.1717
Central East Anatolia	2.2611	1.0253	2.2100	0.0270	0.2515	4.2708
South East Anatolia	0.0522	1.3654	0.0400	0.9690	−2.6240	2.7284
*Constant*	−7.2108	2.6058	−2.7700	0.0060	−12.3182	−2.1035
$$ \mathit{\ln}\left({\sigma}_v^2\right) $$	1.0157	0.2782	3.6500	0.0000	0.4704	1.5610
*exp*(*γ*)/[*exp*(*γ*) + 1]	3.6915	0.2975	12.4100	0.0000	3.1085	4.2745
$$ {\sigma}_S^2={\sigma}_v^2+{\sigma}_v^2 $$	2.7613	0.7683			1.6006	4.7638
$$ \gamma ={\sigma}_u^2/{\sigma}_v^2 $$	0.9757	0.0071			0.9572	0.9863
$$ {\sigma}_u^2 $$	2.6942	0.7672			1.1905	4.1978
$$ {\sigma}_v^2 $$	0.0672	0.0086			0.0503	0.0840

The statistical difference in the inefficiency among hospital types and higher efficiency of MoH General Hospitals can be explained by their high utilization rate (patient volume). Many of the public sector general hospitals are the only source of hospital care in relatively small districts. Moreover, central planning of resource allocation and use of human resources may have affected efficiency levels of these hospitals.

Reported value of γ (0.97) is close to 1 indicating that much of the variation in the composite error term is due to the inefficiency component ([26], p., 250) and only 3% is due to random errors. Hence, hospital inefficiency is highly important in explaining the variability of hospital output.

**Fig. 1:**
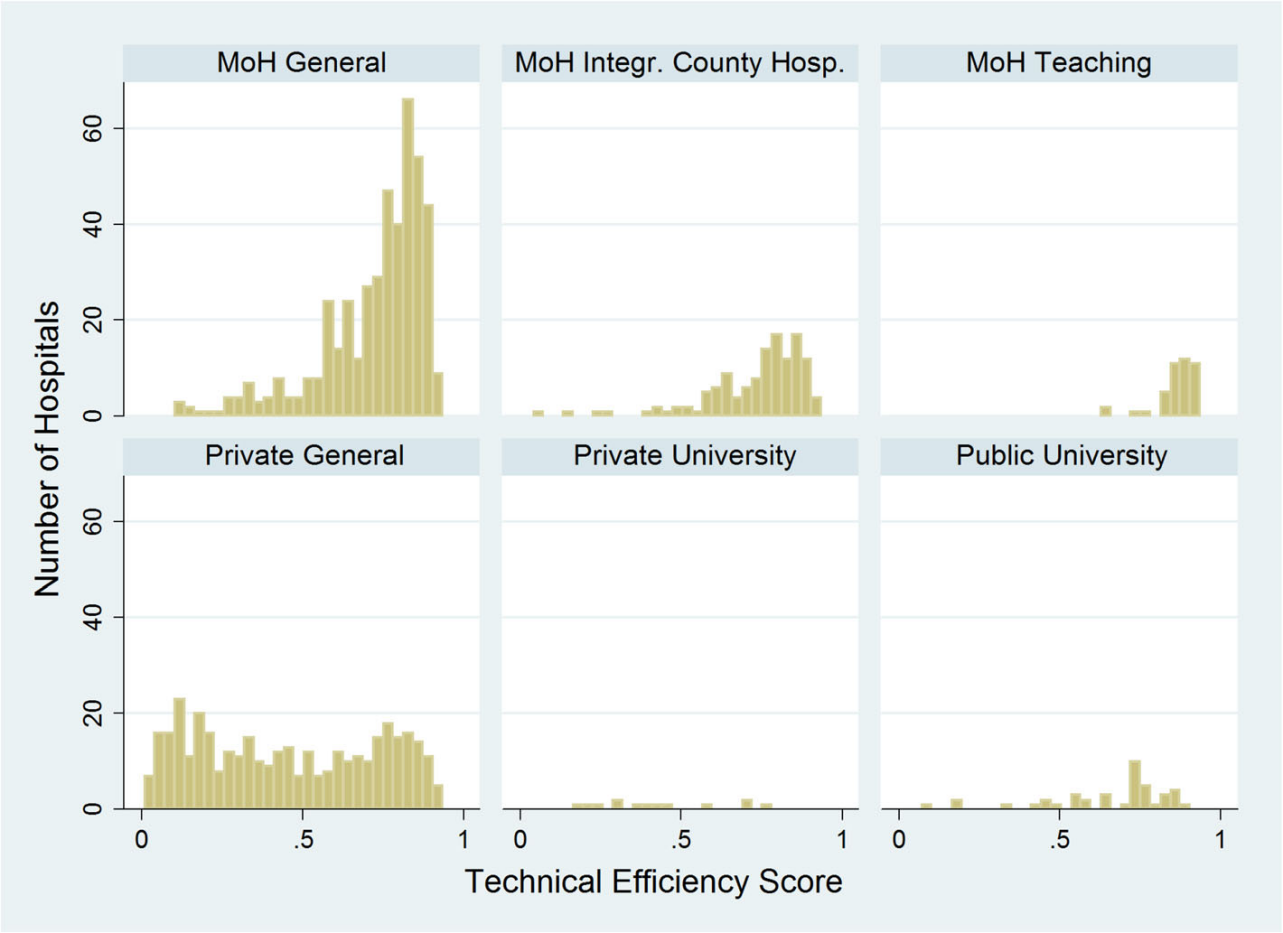
Distribution of Technical Efficiency Scores by Hospital Type

Figure 1 shows the frequency distribution of technical efficiency scores for all six types of hospitals. The efficiency scores of all three MoH Hospitals and Public University Hospitals are skewed towards the right indicating that a higher proportion of these hospitals are among the high efficiency groups. Distribution of efficiency scores for private hospital types show equal distribution along the efficiency plane. The distribution for Private Hospitals shows a distinctive bimodal pattern with wide variability of the scores. Therefore, some of the private hospitals are very efficient while others are very inefficient.

The public and private University hospitals focus significant amount of their resources towards clinical trainings and medical education. Educational mission often requires conducting additional clinical tests and diagnostics for the benefit of learners implying that teaching hospitals are likely to use higher level of resources than non-teaching hospitals for producing the same level of output. Therefore, university hospitals may become less efficient than other general hospitals. A number of research studies also found relatively low efficiency scores for teaching hospitals (e.g. (p. 116) [45–47]). It is interesting that MoH Teaching hospitals show relative high efficiency scores even though teaching and learning functions are important for these hospitals. Unlike the university hospitals, the principal objective of MoH teaching hospitals is to provide hospital services rather than teaching. These hospitals are directly under financial and administrative oversight of the MoH and implementation of cost containment strategies, centralized resource reallocation approach, and other related policies probably helped in improving their efficiency scores.

**Fig. 2:**
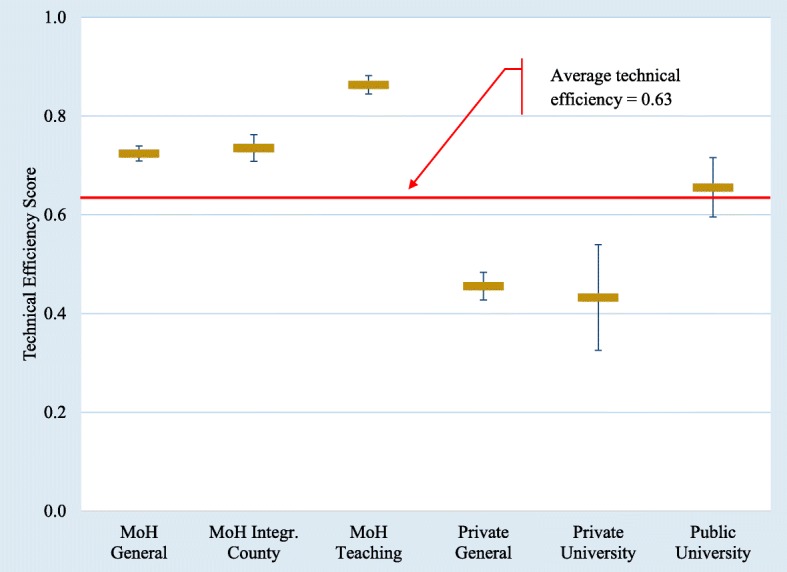
Mean Technical Efficiency Scores and 95% Confidence Interval by Hospital Type

Figure 2 illustrates the average technical efficiency (ATE) scores of various hospital types. The ATE score for all hospitals was 0.63 and ranged from 0.01 to 0.94 with a median of 0.73. About one third of these hospitals had a technical efficiency score between 0.80 and 1.00 and another one third had a score between 0.60 and 0.80. The MoH hospital types reported the highest ATE scores with the MoH Teaching Hospitals leading the group. Public university hospitals follow the MoH hospitals in ATE score. Private hospitals reported the lowest ATE score. Within the MoH hospitals, the Integrated hospitals serve low population density rural provinces. As can be seen from Fig. 2, the integrated hospitals exhibit lower ATE than the MoH Teaching hospitals. This is not surprising because the primary role of these hospitals is to provide basic health services and to function as the social safety net facility in relatively remote and rural areas. These hospitals regularly refer more serious cases to general or teaching hospitals after initial consultation or urgent care. They serve basic surgery needs and non-risk deliveries. They transfers higher risk patients to general hospitals after stabilizing their health condition. The MoH allocates a sufficient number of doctors to these hospitals. However, they are not as ‘busy’ as they would have been in other types of hospitals. Hence, we have a situation where fewer inpatient and outpatient patients are served by greater number of doctors and other healthcare professionals, thus, contributing towards lower efficiency.

Private hospitals report the lowest ATE scores and their technical efficiencies follow a bimodal distribution (Fig. 1). This is also consistent with the nature of private hospital market in Turkey. Private hospitals in Turkey can be subdivided into two types -- smaller hospitals with limited service availability and highly specialized large scale chain hospitals. The lower end of efficiency score distribution among private hospitals represents mainly the small hospitals while the higher efficiency hospitals are the larger comprehensive hospitals. Rural private hospitals tend to fill gaps in hospital services but metropolitan private hospitals serve customers from higher socioeconomic groups. With increasing income of the population, private hospitals are becoming more popular in urban areas and since these hospitals have to compete with MoH general hospitals for patients, remaining efficient is important to maintain or increase the market share.

## Discussion

The relative efficiency scores of hospital types indicate that overall efficiency of hospital sector of Turkey can be improved by encouraging more effective use of resources. In fact, increasing market share of public hospitals will also improve efficiency of resource use. Since the small private hospitals are the least efficient, policy makers should identify strategies to improve efficiency of these hospitals. The integrated public hospitals may be able to improve technical efficiency by becoming better integrated with local private clinics and other hospitals in rural communities.

Affiliation system, which was implemented by the MoH in 2011 [48], facilitates collaboration between University and MoH hospitals by utilizing each other’s resources. Newly established university hospitals have the opportunity to use MOH hospitals’ relatively better infrastructures while MoH hospitals benefit from the expertise and specialization of university hospitals. In the long-run, this approach may help improve efficiency of both Public University and MoH Teaching hospitals. Similar arrangements between MoH, private university, and private hospitals should also be useful in improving efficiency of both private university hospitals and other general private hospitals. Additional efficiency improvements are expected as a result of 2012 reform that established the PHUs through the unification of MoH hospitals of various categories. The unifications are accompanied by implementation of a set of common cost containment and quality control measures, implying that hospitals will become more homogeneous in terms of efficiency.

The reforms initiated by Government of Turkey aimed at improving technical efficiency of all hospitals, especially the secondary and tertiary hospitals. Our findings from 2012 indicate significant variability in the efficiency scores of hospitals. The expectation is that this variability in efficiency scores across hospitals will reduce over the years if the reform initiatives are successful. Our findings may be used as the baseline to evaluate the effect of these hospital sector reforms on hospital efficiency. Future research studies can re-estimate efficiency scores to see how the variability of scores changed over the years with an expectation that the range and variability of hospital efficiency scores will decline over time. The continued reforms are explicitly aiming at improving efficiency by encouraging sharing of resources to maximize hospital production and to improve operational efficiency. If reforms are successful, we should see improvements in overall efficiency scores and reductions in the variability of the scores across hospitals of different types and sizes.

## Conclusions

This study has estimated the technical inefficiency scores for public and private hospitals using a recent and comprehensive hospital dataset available for Turkey. The estimation approach incorporates a number of new empirical aspects for deriving the efficiency scores for multi-product firms. The production and technical efficiency functions are estimated simultaneously and a modification has been introduced to account for zero-value inputs in the logarithmic production function. Assuming that relative prices of various hospital outputs remain more or less constant across hospitals, Hicksian aggregation principle can be used to derive the composite index of output. All significant estimates in the regression model had expected signs. Technical efficiency of hospitals varied across hospital types but not across level of economic development of the region in which the hospitals are located. The analysis also indicates that technical efficiency scores of MoH hospitals are better than those for other hospital types.

It is interesting to note that not all private hospitals are efficient and many are highly inefficient. Small private hospitals are the least efficient hospital category among all the hospital-types in Turkey. The MoH general hospitals were the most efficient hospital category, often better than the urban private hospitals. The efficiency scores of rural MoH hospitals are relatively low but these hospitals are designed as the safety net units in rural areas.

As expected, the University hospitals, both private and public, were less efficient than other non-university hospitals, except for Private General Hospitals. The university hospitals have the important social objective of training health professionals and specialists and therefore, unless the value of medical trainings is taken into account as additional output, these facilities will show low efficiency scores. In that sense, low efficiency scores of university hospitals may not necessarily be interpreted as indication of inefficient use of resources. The values these hospitals create in terms of training may more than offset the additional resources used. Interestingly, the MoH teaching hospitals turned out to be quite efficient in relative terms. The health sector reform in Turkey has emphasized better management of MoH hospitals and it is possible that improved management practices have enhanced relative efficiency scores of MoH teaching hospitals as well.

Turkey considers highly specialized private hospital sector an important component of overall health care system and the results suggest that encouraging establishment of private specialized hospitals will improve overall efficiency. To promote investments in large-scale private hospitals, the Government of Turkey will be creating “health zones” throughout the country to attract foreign direct investment [23, 49].

## Abbreviations

COLS: Corrected Ordinary Least Squares
GDP: Gross Domestic Product
GHI: General Health Insurance
HTP: Health Transformation Program
MLE: Maximum Likelihood Estimates
MoD: Ministry of Defense
MoH: Ministry of Health
OECD: Organization for Economic Cooperation and Development
PHAT: Public Hospitals Administration of Turkey
PHU: Public Hospital Unions
SFA: Stochastic Frontier Analysis
SSI: Social Insurance Institution

## Footnotes

1 A variation of COLS called Modified OLS (MOLS) was proposed by Afrait [50] and Richmond [51], which is very similar to the two-step COLS procedure.

2 $LR=-2\left[\ln \left(L\left({H}_0\right)/L\left({H}_1\right)\right)\right]\sim {\mathrm{X}}_{(n)}^2$ where L(H_0_) is the likelihood value of the restricted estimate, L(H_1_) is the likelihood value of for the unrestricted estimate, and *n* is the number of restrictions imposed by the null hypothesis [40].

3 LR = 408; $$ {\mathrm{X}}_{(21)}^2=32.67 $$.
